# Development of an experiment-split method for benchmarking the generalization of a PTM site predictor: Lysine methylome as an example

**DOI:** 10.1371/journal.pcbi.1009682

**Published:** 2021-12-08

**Authors:** Guoyang Zou, Yang Zou, Chenglong Ma, Jiaojiao Zhao, Lei Li

**Affiliations:** 1 School of Basic Medicine, Qingdao University, Qingdao, China; 2 College of Life Science, Qingdao University, Qingdao, China; 3 School of Data Science and Software Engineering, Qingdao University, Qingdao, China; University of Calgary, CANADA

## Abstract

Many computational classifiers have been developed to predict different types of post-translational modification sites. Their performances are measured using cross-validation or independent test, in which experimental data from different sources are mixed and randomly split into training and test sets. However, the self-reported performances of most classifiers based on this measure are generally higher than their performances in the application of new experimental data. It suggests that the cross-validation method overestimates the generalization ability of a classifier. Here, we proposed a generalization estimate method, dubbed experiment-split test, where the experimental sources for the training set are different from those for the test set that simulate the data derived from a new experiment. We took the prediction of lysine methylome (Kme) as an example and developed a deep learning-based Kme site predictor (called DeepKme) with outstanding performance. We assessed the experiment-split test by comparing it with the cross-validation method. We found that the performance measured using the experiment-split test is lower than that measured in terms of cross-validation. As the test data of the experiment-split method were derived from an independent experimental source, this method could reflect the generalization of the predictor. Therefore, we believe that the experiment-split method can be applied to benchmark the practical performance of a given PTM model. DeepKme is free accessible via https://github.com/guoyangzou/DeepKme.

## Author summary

The performance of a model for predicting post-translational modification sites is commonly evaluated using the cross-validation method, where the data derived from different experimental sources are mixed and randomly separated into the training dataset and validation dataset. However, the performance measured through cross-validation is generally higher than the performance in the application of new experimental data, indicating that the cross-validation method overestimates the generalization of a model. In this study, we proposed a generalization estimate method, dubbed experiment-split test, where the experimental sources for the training set are different from those for the test set that simulate the data derived from a new experiment. We took the prediction of lysine methylome as an example and developed a deep learning-based Kme site predictor DeepKme with outstanding performance. We found that the performance measured by the experiment-split method is lower than that measured in terms of cross-validation. As the test data of the experiment-split method were derived from an independent experimental source, this method could reflect the generalization of the prediction model. Therefore, the experiment-split method can be applied to benchmark the practical prediction performance.

This is a *PLOS Computational Biology* Benchmarking paper.

## Introduction

Protein lysine methylation, as one type of dynamic and reversible post-translational modifications (PTMs) by protein lysine methyltransferases and demethylases, plays an important role in cell signaling and regulation [1]. This modification contains three different types: mono-, di- and tri-methylation (i.e. Kme1, Kme2 and Kme3). The majority of Kme sites are discovered through the combination of affinity purification and high-throughput mass spectrometry. Besides those identified by experiments, a bunch of computational approaches were developed for the prediction of Kme sites. A few predictors were based on Support Vector Machine (SVM) combined with different features, such as intrinsic disorder information [2] or linear functional motif as the feature [3]. Recently, a few predictors [4,5] were based on deep-learning (DL) algorithms. Cross-validation is the general method to evaluate prediction models using a limited data set. This data set is commonly composed of experimental data from different sources and randomly split into training and validation sets. The cross-validation evaluation is often considered the measure of the generalization ability. However, it is found that the self-reported performance, which was documented in the original literature calculated in terms of cross-validation and/or the independent test, overestimates the real accuracy based on newly constructed independent datasets [6–8]. It indicates that the self-reported performance may not be indicative of prediction quality. Therefore, experimentalists should be careful to use PTM predictors and independent assessments are necessary to evaluate their performances in practice [7,8].

Here, we proposed a method for generalization estimation, called the experiment-split test, to benchmark models for their practical performances. In this method, the data of the training and test sets are derived from different experiments and the common data between both sets are removed from the test set so that both sets are independent. Therefore, the test set simulates a newly constructed independent dataset. To evaluate this novel method, we took the prediction of lysine methylome (Kme) as an example. We developed a DL-based predictor DeepKme with superior performance to existing methods. We found that the performance measured using cross-validation was larger than that measured using the experiment-split test. As the test set in the experiment-split method is derived from an independent experimental source, the experiment-split performance reflects the generalization ability of the predictor. DeepKme is free accessible via https://github.com/guoyangzou/DeepKme.

## Methods

### Dataset construction and pre-processing

**Fig 1:**
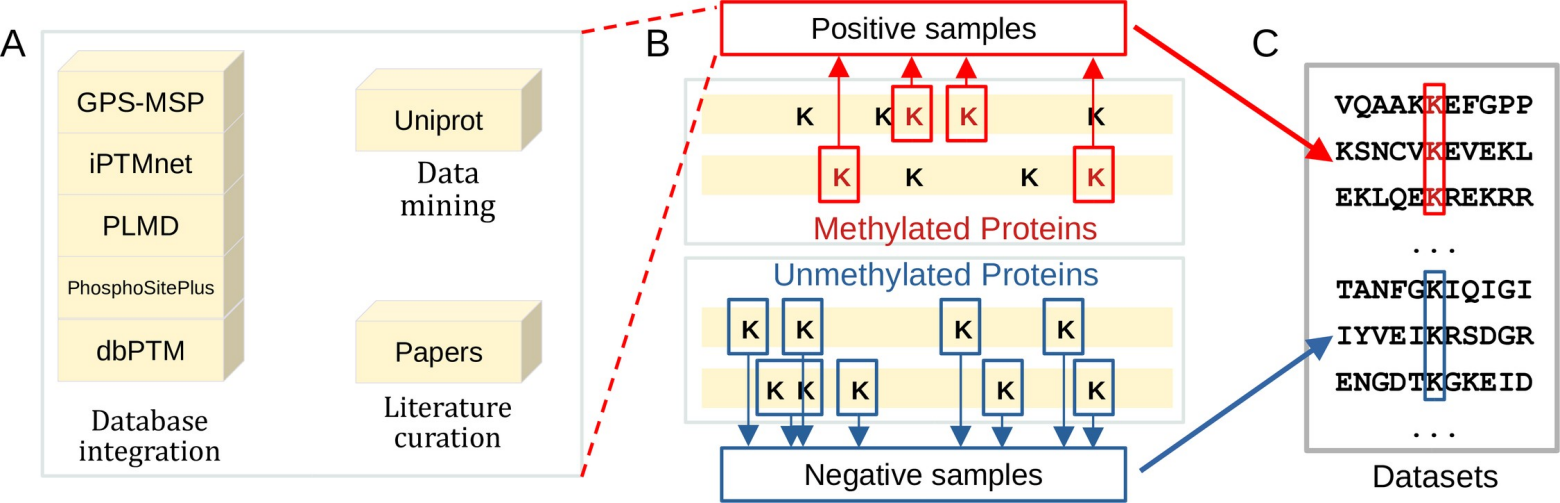
The working flow of data collection.

The data about lysine methylation sites were collected through three approaches: database integration, data mining, and literature curation (Fig 1A), which include GPS-MSP [9], iPTMnet [10], PLMD [11], PhosphoSitePlus [12], dbPTM [13], UniProt [14] and literature [15]. We initially collected 5450 Kme sites from 2989 human proteins and all the sites were annotated with the original experimental sources (S1 and S2 Tables). We used a sequence window of 61 amino acids in length with “K” in the center to represent the site. If the central lysine residue is located near the N-terminus or C-terminus of the protein sequence, the symbol “X” is added at the related terminus to ensure the window sizes of the sequences are the same. After removing the replicates, 5229 Kme sequences were retained (Fig 1B). Four different labels (i.e. Kme1, Kme2, Kme3 and Kme) were assigned to each sequence if the sequence was modified by lysine mono-, di-, tri-methylation or methylation. Moreover, we collected 638,805 lysine sites without methylation annotations from human proteome as negative samples and their related sequences were unique and different from the positive sequences (Fig 1B and 1C).

### Experiment-split method

**Fig 2:**
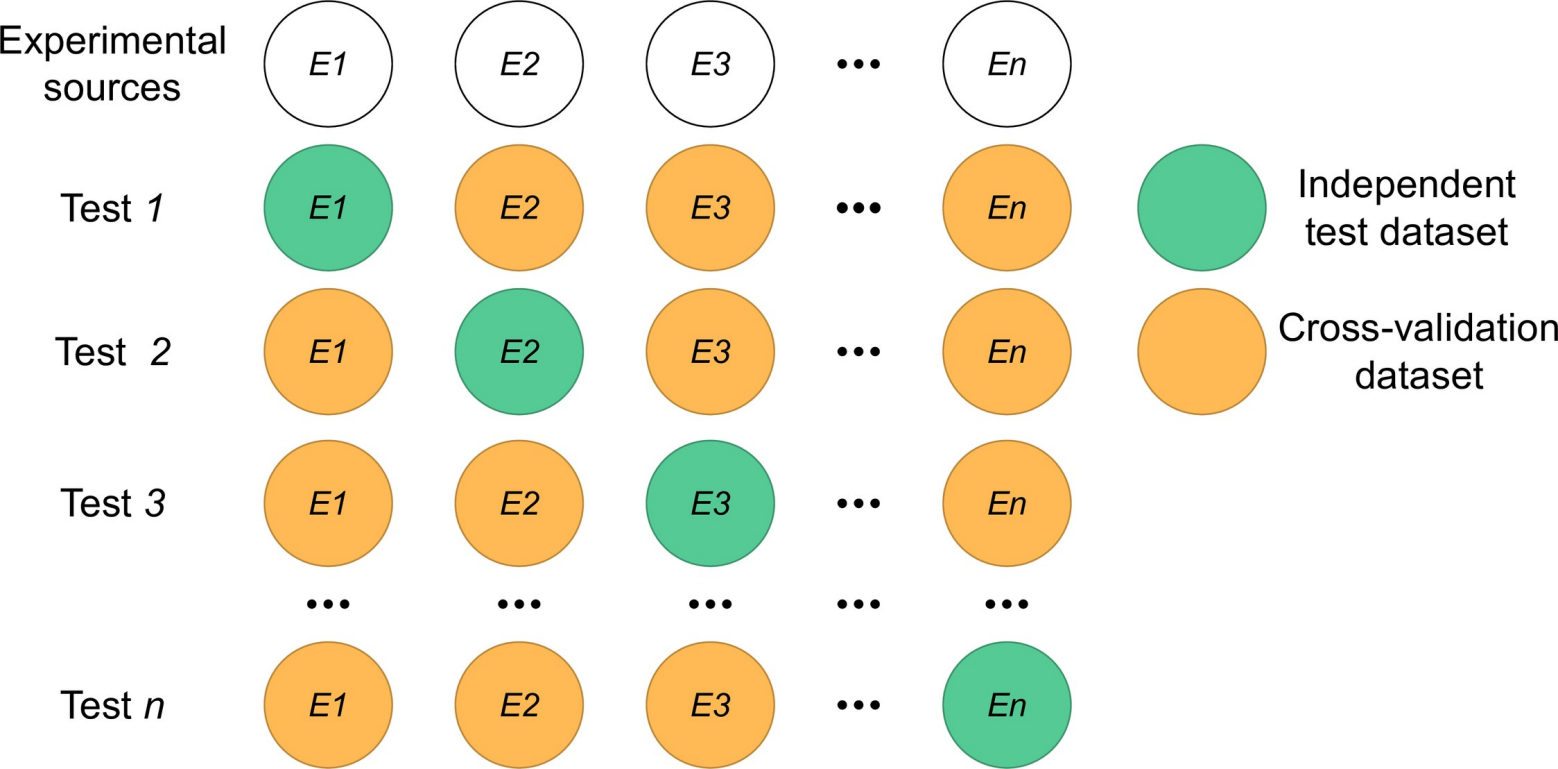
Illustration of the experiment-split method. *En* represents the data from the *n*th experimental source. Test *n* represents that the *En* data is used for the independent test and the rest experimental data for the training.

Fig 2 illustrates the experiment-split test method. For instance, we collected data from *n* different experimental sources and therefore we could make the tests *n* times. In test *i*, the PTM data from the experimental source *i* were used as positives of the independent test dataset; the data from the rest experimental sources were considered the positive samples in the training dataset. It should be noted that the common data between the training and test sets are removed from the test set so that both sets are independent. For convenience and the consideration of computational cost, we randomly chose 40000 samples from all the non-PTM-containing proteins as negatives and split them into half, one for training and the other for testing. We reason that the performance estimation may be unreliable if the number of positive samples in the test set is extremely small or few test sets are available. Therefore, we balanced these two numbers. In this study, we evaluated the prediction performance based on at least five test sets and each containing at least five positive samples.

### Feature encodings

#### One-Hot (OH) encoding

It is represented by the conversion of the 20 types of amino acids to 20 binary bits. By considering the complemented symbol “X”, 21 (= 20+1) binary bits are used to represent a single position in the peptide sequence (S1 Fig). For example, the amino acid “Q” is represented by “100000000000000000000” and “H” is represented by “000000000000000000010”.

#### Position-Specific Scoring Matrix (PSSM) encoding

It is generated through running the PSI-BLAST program and described elsewhere [16,17].

#### Word Embedding (WE) encoding

Each item of the input sequence is encoded by One-Hot encoding to a 21-dimension binary vector, followed by a fully connected layer without nonlinear activation function which is used to decrease the vector to a five-dimension vector.

### Model construction

#### The 1D-CNN Model with OH Encoding (CNN_OH_)

**Fig 3:**
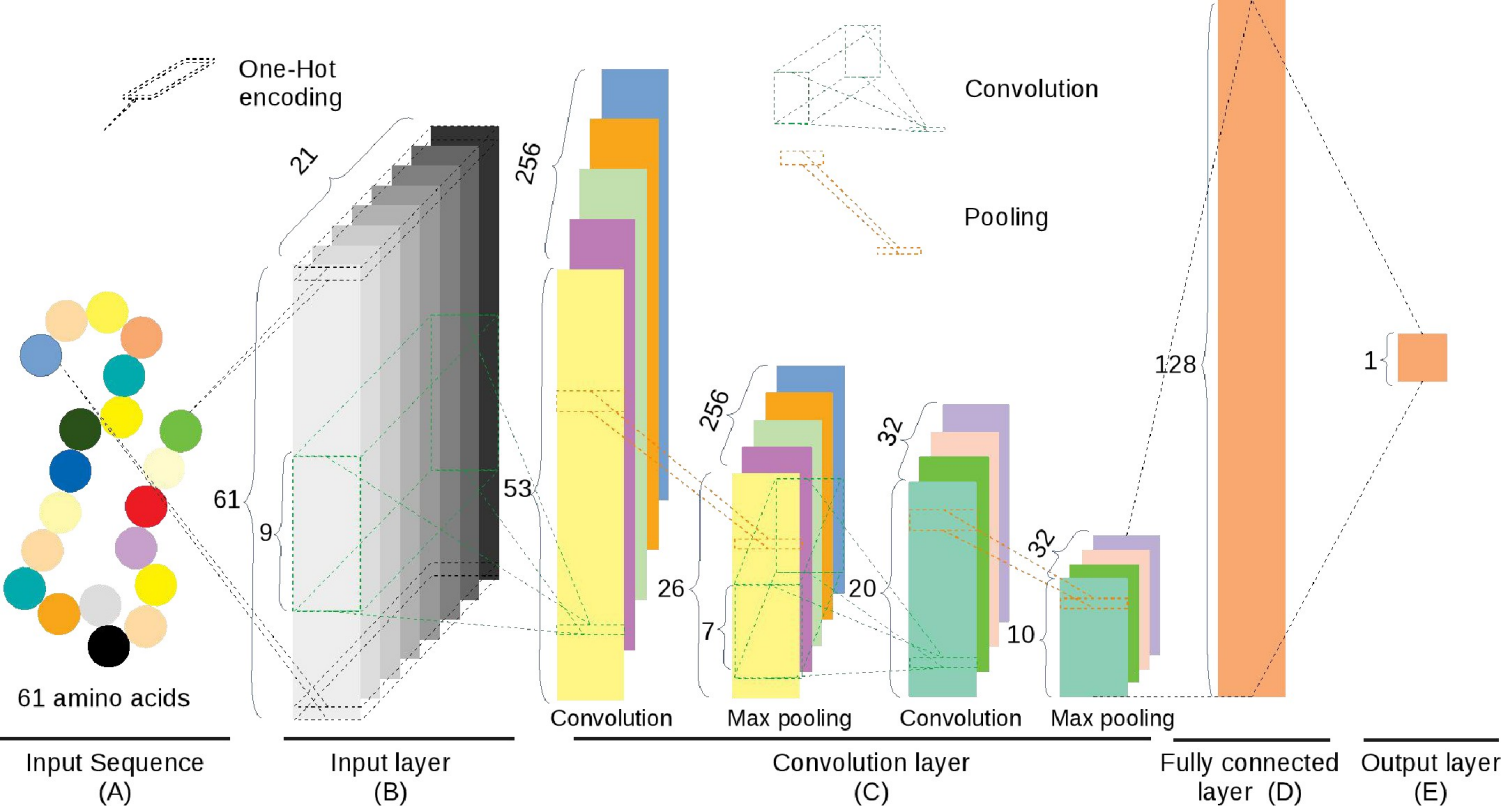
The graph representation of the CNN_OH_ model. (A) The input sequence consists of 61 amino acids. (B) In the input layer, the input sequence is represented by a binary matrix using the One-Hot encoding. (C) The convolution layer contains two convolution sublayers and two max-pooling sublayers. D) Fully connected layer. The output matrix from the convolution layer is nonlinearly transformed to 128 representative features. E) Output layer. The modification score is calculated based on the 128 features. The details are described in the Methods section.

This model contains four layers, listed below (Fig 3).

1. Input layer. Each input sequence of 61 amino acids is encoded by the OH encoding to a 61×21 binary matrix.
2. Convolution layer. It consisted of two convolution sublayers, each followed by individual max-pooling sublayers, respectively. The first convolution sublayer includes 256 different convolution kernels with the size of 9×21. Each kernel is applied to the 61×21 matrix from the input layer and results in a feature vector with the size of 53 (= 61–9+1). Thus, the 256 kernels output a 53×256 matrix. Next, a pooling kernel with the size of 2 is applied to the feature matrix and produces a 26×256 matrix. In the second convolution sublayer, 32 different convolution kernels with the size 7×256 are applied to generate a 20×32 matrix, followed by a pooling kernel with size 2 that produces a 10×32 data matrix.
3. Fully connected layer. The 10×32 data matrix generated from the convolution layer is nonlinearly transformed to 128 representative features.
4. Output layer. The modification score is calculated based on the 128 features using the ‘Sigmoid’ function.

#### The 1D-CNN Model with PSSM Encoding (CNN_PSSM_)

It is similar to CNN_OH_ except that the encoding approach is changed from OH to PSSM.

#### The 1D-CNN Model with WE layer (CNN_WE_)

It is similar to CNN_OH_ except that a fully connected layer is added behind the input layer of CNN_OH_ that converts the 21-dimension binary vector into a five-dimension WE vector.

#### The LSTM Model with OH Encoding (LSTM_OH_)

**Fig 4:**
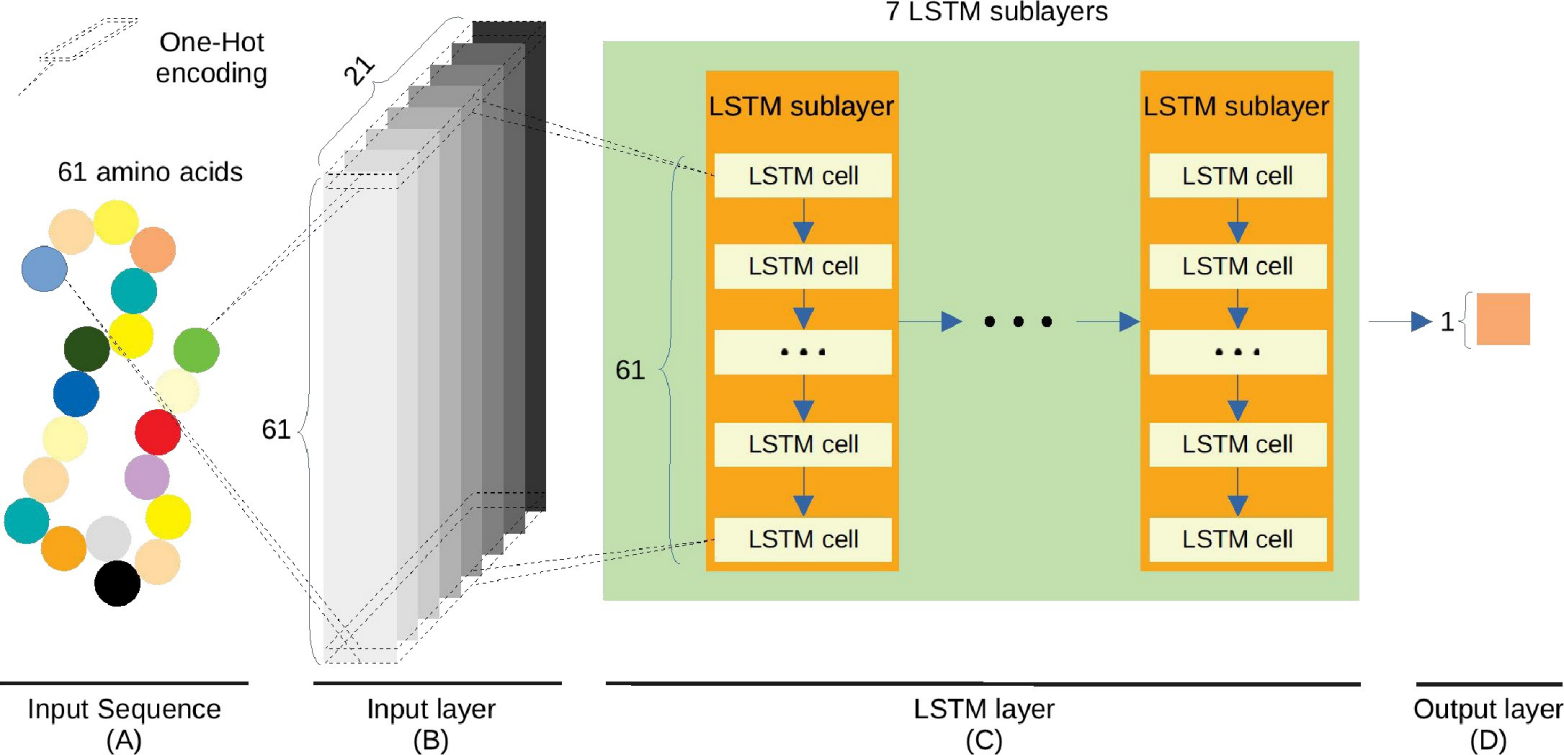
Graph representation of the LSTM_OH_. A) The input sequence consists of 61 amino acids. B) In the input layer, the sequence is represented by a 61×21 matrix through the One-Hot encoding. C) The LSTM layer includes seven LSTM sublayers. Every sublayer contains 61 sequentially connected LSTM cells, each of which contains 32 hidden neuron units. The output data from the former LSTM sublayer are fed to the latter LSTM sublayer. D) Output layer. The output from the LSTM layer is used to calculate the modification score.

This model contains three layers (Fig 4).

1. Input layer. The sequence is represented by a 61×21 matrix through the OH encoding.
2. LSTM layer. It includes seven LSTM sublayers. Every sublayer contains 61 sequentially connected LSTM cells, corresponding to the 61 amino acids of the input sequence. Each LSTM cell contains 32 hidden neuron units and outputs a vector with the size of 32. Every cell is used to process the information from the corresponding amino acid and the upstream LSTM cell. Next, 61 vectors outputted from the first LSTM sublayer are fed to the next LSTM sublayer. The same process is replicated until the last LSTM sublayer. Lastly, the vector from the 61st LSTM cell in the 7th LSTM sublayer is regarded as the output of the LSTM layer to represent the features of the input peptide sequence.
3. Output layer. The vector of 32 features from the LSTM layer is used to calculate the modification score through the “Sigmoid” function.

#### The LSTM Model with PSSM Encoding (LSTM_PSSM_)

It is similar to LSTM_OH_ except that the encoding method is changed from OH to PSSM.

#### The LSTM Model with the WE layer (LSTM_WE_)

It is similar to LSTM_OH_ except that a fully connected layer is added behind the input layer of CNN_OH_ that converts the 21-dimension binary vector into a five-dimension WE vector.

#### The GRU Models with OH Encoding (GRU_OH_), PSSM Encoding (GRU_PSSM_) or the WE layer (GRU_WE_)

The models are similar to the corresponding LSTM models except that the LSTM cells are replaced by the GRU cells.

### The strategy of avoiding overfitting

The parameters in the DL models are trained and optimized based on binary cross-entropy loss function using the Adam algorithm. The maximum of the training cycles is set through the optimized number of epochs to ensure that the loss function value converged. In each epoch, the training dataset is separated with the batch size as 512 and iterated. To avoid overfitting, the early-stopping strategy is applied, where the training process is stopped early when the training loss does not go down within 25 consecutive iterations. The model with the smallest training loss is saved as the best model. Moreover, the dropout rates of the two CNN layers are set at 0.5 and 0.7 respectively, which are obtained through manual hyperparameter optimization.

## Results

### Existing Kme models evaluated using new data showed overestimation

**Fig 5:**
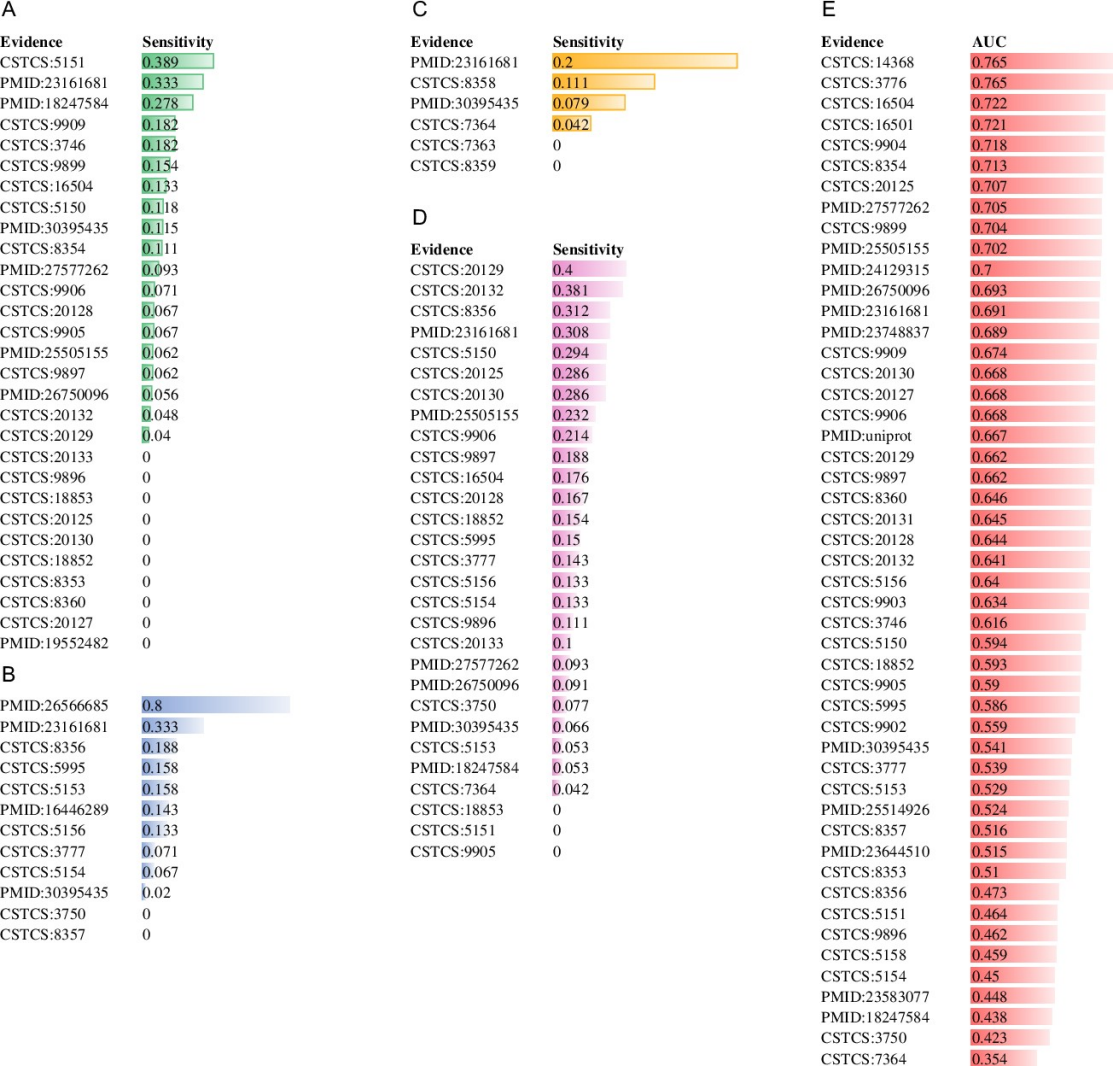
Performance of GPS-MSP and MusiteDeep assessed using different experimental sources. It included the GPS-MSP prediction performances for Kme1 (A), Kme2 (B), Kme3 (C) and Kme (D), and the MusiteDeep performance for Kme (E).

Most PTM predictors are measured using cross-validation but the blind assessment are not generally performed. Here, we took lysine methylome as the study case and investigated the reported Kme classifiers GPS-MSP and MusiteDeep [5] using multiple experimental sources as the test sets, which were independent of the training datasets of the models. The number of experimental sources varies according to the number of sources used for the model training. For instance, 29 different sources were used as the test sets to estimate the performance of the GPS-MSP Kme model whereas 49 distinct sources were selected for MusiteDeep. In addition, the common data between the training set and the test set were discarded from the test set. As GPS-MSP provided the predicted sensitivity value when the specificity value was set as 0.9, we fixed the specificity value as 0.9 as well for the independent test and used the same data preprocessing for the GPS-MSP construction. We performed the tests for all the four modification models and the sensitivity values were significantly lower than the self-reported values (Tables 1 and S3 and Fig 5), suggesting that the self-reported performance of GPS-MSP was overestimated. In addition, since the MusiteDeep performance was assessed using the AUC value, we used the AUC value to estimate its performance. Our calculated mean AUC value (0.606) is significantly smaller than the reported value (0.951; P = 0, single-sample t-test; Tables 1 and S3 and Fig 5). These two analyses indicate that the self-reported performance fails to represent the generalization ability. This caused our interest to develop a method for generalization estimation. It should be noted that GPS-MSP was designed to predict both lysine and arginine methylation sites and it may have a good prediction performance for arginine methylation sites.

**Table 1:** The comparison between evaluated performances of GPS-MSP and MusiteDeep and their self-reported performances.

GPS-MSP				
Type	Number of test datasets^a^	Sn (tested in this study)^b^	Sn (reported)^b^	P value^d^
Kme1	29	0.088±0.103^c^	0.466 [11]	0
Kme2	12	0.173±0.219^c^	0.422 [11]	0
Kme3	6	0.072±0.076^c^	0.764 [11]	0
Kme	29	0.160±0.113^c^	0.445 [11]	0
MusiteDeep				
Type	Number of test datasets	AUC (tested in this study)	AUC (reported)	P value^d^
Kme	49	0.606±0.103^c^	0.951 [6]	0

^a^Test datasets are derived from different experimental sources

^b^Sensitivity value when specificity was set 0.9

^c^These values represent the average and standard deviation (SD), respectively

^d^P-value was calculated using a single-sample t-test.

### CNN_OH_ and CNN_PSSM_ performed best in the constructed models

Computational approaches for predicting PTM sites are based on different algorithms and various predefined characteristics. Generally, the RF and SVM algorithm shows comparable prediction performance in traditional machine-learning (ML) algorithms [18,19]. Deep-learning algorithms have been widely used in PTMs prediction and demonstrated better performances than traditional ML algorithms [4,20–24]. Therefore, we only constructed and compared DL models for Kme prediction.

**Fig 6:**
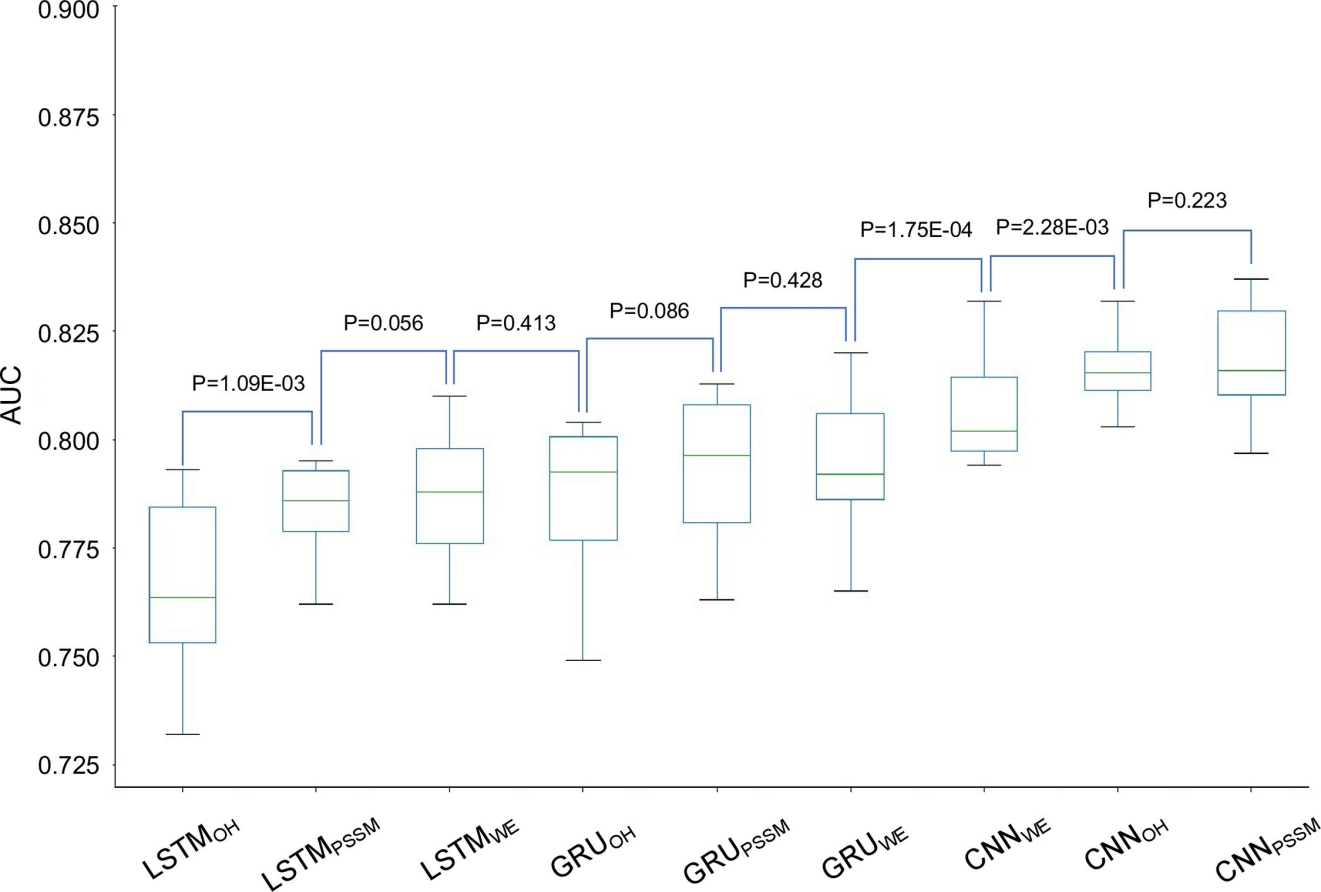
The performances of different DL models for the prediction of Kme1 sites using ten-fold cross-validation.

We collected 4423 Kme1 sites, 635 Kme2 sites, 419 Kme3 sites and 5450 Kme sites from different sources (Fig 1). We constructed ten different DL models with distinct DL architectures and encoding approaches, e.g. CNN_OH_, LSTM_WE_ and GRU_PSSM_ (see Methods for details). Here, we selected the Kme1 type as the study case with the same number of positive and negative samples and constructed the related classifiers and compared their performances in terms of ten-fold cross-validation. The AUC values of CNN_OH_ and CNN_PSSM_ were similar (AUC = 0.817, P = 0.223) and significantly larger than those of other classifiers (P<2.28×10^−3^) (Fig 6). Therefore, we selected CNN_OH_ to construct the model DeepKme for the prediction of Kme1/Kme2/Kme3/Kme sites. The average AUC values of DeepKme for Kme1/Kme2/Kme3/Kme were 0.8355/0.7002/0.7579/0.8062 using ten-fold cross-validation, respectively.

### Evaluation of generalization ability using experiment-split test and comparison with cross-validation

Most if not all the models developed before are assessed in terms of cross-validation and/or independent test. The datasets of cross-validation and the independent test are a mixture of different experimental sources. Although the validation set and the independent set are different from the training set, they are not from the independent experimental source. This may be the main reason why the models fail to reach the reported performances in practice. Here, we developed the experiment-split method for estimating generalization (see Methods for detail). This method is based on the fact that there are multiple experimental sources and each of them can be considered the independent test set to estimate the performance. We performed 27/12/9/40 independent tests for the Kme1/Kme2/Kme3/Kme models, respectively. The mean AUC values for these models is 0.766, 0.660, 0.729 and 0.747, respectively (Table 2). Specifically, the AUC values for the Kme1/Kme models are smaller than the corresponding AUC values calculated based on ten-fold cross-validation (P = 0.018 or 0.013), whereas the AUC values for the Kme2/Kme3 models are similar to the AUC values in terms of cross-validation (P = 0.16 or 0.44) (Table 2). These comparisons indicate that the experiment-split method is the better measure of the generalization for the Kme1/Kme models than cross-validation, whereas both measures are comparable for the Kme2/Kme3 models. Additionally, the standard deviation (SD) values of the cross-validation performances are narrower than those of the experiment-split performances (P = 1.83E-2, paired t-test; Table 2). For instance, the SD value of the former for the Kme2 model is smaller than 0.03 while that of the latter is larger than 0.08. It suggests that the data from different experimental sources are divergent and the mixture of these sources in cross-validation reduces the data diversity.

**Table 2:** Performance comparison of CNN_OH_ models between cross-validation and experiment-split test.

Modification type	10-fold cross-validation^a^	Experiment-split^a^	P value^b^
**Kme1**	0.836±0.011	0.766±0.141	0.018
**Kme2**	0.700±0.026	0.660±0.088	0.16
**Kme3**	0.758±0.039	0.729±0.096	0.44
**Kme**	0.806±0.012	0.747±0.140	0.013

^a^Average and SD of the AUC values

^b^P-value was calculated using paired t-test.

We compared the performances of GPS-MSP, MusiteDeep and our CNN_OH_ model using the experiment-split method. As the three models are constructed using different training data and the data from the experimental sources for testing need to be independent of each training data, the test datasets for each model may be different and positive samples from the same experimental sources may also be distinct. Therefore, the construction of the test sets is complex compared to the construction of traditional cross-validation and independent datasets. Despite it, we reason that their performances can be fairly compared using statistical analysis. We collected the AUC values for the three models calculated using the experimental-split method (S3 and S4 Tables) and summarized them in Table 3. The AUC values of the CNN_OH_ models are statistically larger than those of the GPS-MSP and MusiteDeep models (Table 3). Therefore, the CNN_OH_ models have outstanding generation ability.

**Table 3:** Comparison of experiment-split performances for the models.

Modification type	CNN_OH_^a^	GPS-MSP^a^	P value^b^
**Kme1**	0.766±0.143	0.568±0.079	3.13E-8
**Kme2**	0.660±0.092	0.565±0.118	0.039
**Kme3**	0.729±0.102	0.515±0.092	4.48E-3
**Kme**	0.747±0.141	0.539±0.082	9.42E-10
	**CNN**_**OH**_ ^**a**^	**MusiteDeep** ^**a**^	**P value** ^**b**^
**Kme**	0.747±0.141	0.606±0.103	2.11E-3

^a^Average and SD of the AUC values

^b^P-value was calculated using the student’s t-test.

## Discussion and conclusions

Cross-validation is the common resampling technique to evaluate machine-learning models constructed using a limited amount of samples. It is used to assess the generalization of a predictive model to independent data sets and estimate the practical accuracy of a predictive model. Nevertheless, based on newly constructed independent datasets, the cross-validation performance is repeatedly found to overestimate the real accuracy measured on independent datasets [6–8]. For example, 11 online programs for the prediction of four lysine PTM types (i.e. acetylation, methylation, SUMOylation and ubiquitination) were assessed and nine of them performed close to random [8]. To further estimate the reported performance in literature, we tested two models (GPS-MSP [9] and MusiteDeep [5]) using different experimental sources. GPS-MSP was designed to predict lysine and arginine PTM sites based on the traditional machine-learning algorithm whereas MusiteDeep was developed to predict the sites of multiple PTM types based on a deep-learning algorithm. We found that the performances of both models in terms of the independent test were lower than the self-reported performances. This observation is consistent with the previous observations [6–8].

To find the proper measure indicative of prediction quality in practice, we developed the experiment-split method. This method requires numerous experimental sources so that each source can be considered the independent test dataset. We took lysine methylome as the study case because of a variety of experimental sources available. We constructed four CNN_OH_ models corresponding to the prediction of Kme1/Kme2/Kme3/Kme, respectively. We found that the experiment-split performances of the Kme1/Kme models were smaller than the related cross-validation performances, whereas the experiment-split performances for the Kme2/Kme3 models were similar to those evaluated using the cross-validation. As the test set of the experiment-split method is the data from an independent experimental source, the experiment-split measure could reflect the generalization ability of a model.

**Fig 7:**
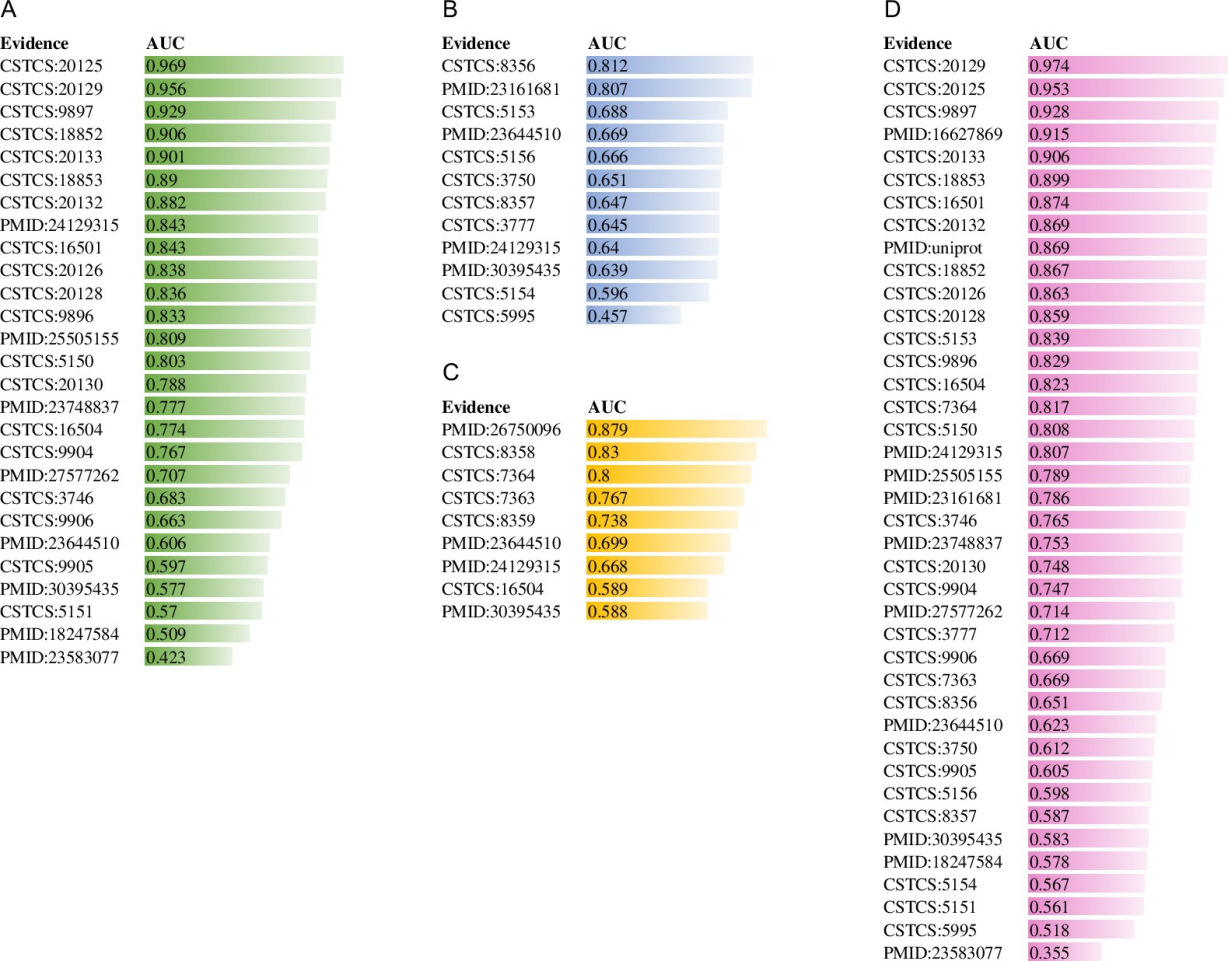
The CNN_OH_ performances were assessed by the experiment-split method. The performances of the CNN_OH_ model for Kme1 (A), Kme2 (B), Kme3 (C) and Kme (D) were evaluated using various independent experimental sources, respectively.

Although the experiment-split method is suitable to assess the generation ability of a prediction model, it has several disadvantages. First, it requires a variety of experimental sources. The more the number of experimental sources, the more reliable the experiment-split performance. Second, different experimental sources are not uniform in size and the performance of the model built based on a small training dataset may be lower than that of the model constructed using a large training dataset. Therefore, the experimental sources with big PTM data are suitable to be considered part of the training set rather than the test set. We suggest here that the independent test data set should occupy less than 1/5 of the total collected data. Third, the experiment-split performances are diverse for different experiment sources, suggesting the difficulty in reliably estimating the prediction performance for a given experiment. It is true since the PTMs in the different cells or tissues are catalyzed by different enzymes with diverse characteristics and the PTMs identified from these cells or tissues have distinct features. If the data set to be predicted contains the information included in the training set, the developed model may show good prediction performance; otherwise, the performance seems poor. The suggested solution for this disadvantage is the collection of more experimental sources for testing and statistical analyses need to be used for the estimation. Although the experiment-split method has these drawbacks, this method is reliable to estimate the generalization of a predictor compared to cross-validation (Fig 7 and Table 2).

## Supporting information

S1 Table Summary of the data size from different resources. (DOCX)

S2 Table Summary of the data size from different experimental sources. (DOCX)

S3 Table Prediction performance for MSP and MusiteDeep in terms of the experiment-split test. (DOCX)

S4 Table Prediction performance for CNN_OH_ in terms of the experiment-split test. (DOCX)

S1 Fig Illustration of the One-Hot encoding. (TIF)
