# Supplementary material for: Development of an experiment-split method for benchmarking the generalization of a PTM site predictor: Lysine methylome as an example
Source: PLoS Comput Biol. 2021 Dec 8;17(12):e1009682. doi: 10.1371/journal.pcbi.1009682 (PMC8687584; doi:10.1371/journal.pcbi.1009682)
Supplement: S1 Table — (DOCX) [file pcbi.1009682.s001.docx]

**S1 Table. Summary of the data size from different resources.**

|  | **GPS-MSP** | **iPTMnet** | **PLMD** | **PhosphoSitePlus** | **dbPTM** | **UniProt** | **Literature** | **All** |
| --- | --- | --- | --- | --- | --- | --- | --- | --- |
| **Kme1** | 279 | 0 | 0 | 4252 | 0 | 175 | 54 | 4423 |
| **Kme2** | 107 | 0 | 0 | 529 | 0 | 65 | 53 | 635 |
| **kme3** | 56 | 0 | 0 | 326 | 0 | 59 | 65 | 419 |
| **Kme** | 695 | 210 | 1901 | 5004 | 3431 | 228 | 172 | 5450 |
