# Supplementary material for: Development of an experiment-split method for benchmarking the generalization of a PTM site predictor: Lysine methylome as an example
Source: PLoS Comput Biol. 2021 Dec 8;17(12):e1009682. doi: 10.1371/journal.pcbi.1009682 (PMC8687584; doi:10.1371/journal.pcbi.1009682)
Supplement: S2 Table — (DOCX) [file pcbi.1009682.s002.docx]

**S2 Table. Summary of the data size from different experimental sources.**

| **Index** | **Experimental source** | **Number of modification sites** |
| --- | --- | --- |
| **1** | PMID:25514926 (database) | 3296 |
| **2** | PMID:26750096 | 2116 |
| **3** | PMID:23644510 | 1644 |
| **4** | PMID:24129315 | 991 |
| **5** | PMID:23748837 | 441 |
| **6** | PMID:25505155 | 396 |
| **7** | PMID:30395435 | 344 |
| **8** | PMID:uniprot | 274 |
| **9** | CSTCS:20129 | 187 |
| **10** | CSTCS:9897 | 187 |
| **11** | CSTCS:20128 | 172 |
| **12** | CSTCS:20132 | 169 |
| **13** | CSTCS:20133 | 149 |
| **14** | CSTCS:18852 | 149 |
| **15** | CSTCS:18853 | 149 |
| **16** | PMID:23583077 | 136 |
| **17** | PMID:16627869 | 131 |
| **18** | CSTCS:20130 | 130 |
| **19** | CSTCS:20126 | 116 |
| **20** | CSTCS:20125 | 116 |
| **21** | PMID:27577262 | 115 |
| **22** | CSTCS:16504 | 112 |
| **23** | CSTCS:20127 | 104 |
| **24** | CSTCS:20131 | 99 |
| **25** | PMID:17194708 | 98 |
| **26** | PMID:23161681 | 96 |
| **27** | CSTCS:9896 | 88 |
| **28** | CSTCS:16501 | 76 |
| **29** | CSTCS:9905 | 68 |
| **30** | PMID:18438403 | 68 |
| **31** | PMID:16267050 | 64 |
| **32** | CSTCS:9906 | 64 |
| **33** | CSTCS:9904 | 62 |
| **34** | CSTCS:7364 | 61 |
| **35** | CSTCS:5150 | 59 |
| **36** | CSTCS:9899 | 58 |
| **37** | CSTCS:5153 | 53 |
| **38** | CSTCS:5995 | 53 |
| **39** | CSTCS:8353 | 50 |
| **40** | CSTCS:8356 | 49 |
| **41** | CSTCS:5151 | 48 |
| **42** | CSTCS:3750 | 46 |
| **43** | CSTCS:9903 | 45 |
| **44** | CSTCS:5156 | 42 |
| **45** | CSTCS:3746 | 41 |
| **46** | PMID:18247584 | 39 |
| **47** | CSTCS:5154 | 39 |
| **48** | CSTCS:9902 | 38 |
| **49** | CSTCS:3777 | 38 |
| **50** | CSTCS:5996 | 37 |

Different experimental sources have duplicated data.
