# Supplementary material for: Development of an experiment-split method for benchmarking the generalization of a PTM site predictor: Lysine methylome as an example
Source: PLoS Comput Biol. 2021 Dec 8;17(12):e1009682. doi: 10.1371/journal.pcbi.1009682 (PMC8687584; doi:10.1371/journal.pcbi.1009682)
Supplement: S3 Table — (DOCX) [file pcbi.1009682.s003.docx]

**S3 Table. Prediction performance for MSP and MusiteDeep in terms of experiment-split test.**

| **Predictor** | **Type** | **Number** | **Experimental source** | **AUC** | **Sensitivity*** |
| --- | --- | --- | --- | --- | --- |
| **GPS-MSP** | **Kme1** | 1407 | PMID:26750096 | 0.484 | 0.056 |
|  |  | 211 | PMID:25505155 | 0.54 | 0.062 |
|  |  | 75 | PMID:27577262 | 0.547 | 0.093 |
|  |  | 52 | PMID:30395435 | 0.588 | 0.115 |
|  |  | 32 | CSTCS:9897 | 0.504 | 0.062 |
|  |  | 30 | CSTCS:20128 | 0.551 | 0.067 |
|  |  | 25 | CSTCS:20129 | 0.591 | 0.04 |
|  |  | 21 | CSTCS:20132 | 0.585 | 0.048 |
|  |  | 20 | CSTCS:20133 | 0.604 | 0 |
|  |  | 18 | PMID:18247584 | 0.634 | 0.278 |
|  |  | 18 | CSTCS:5151 | 0.672 | 0.389 |
|  |  | 18 | CSTCS:9896 | 0.519 | 0 |
|  |  | 18 | CSTCS:18853 | 0.552 | 0 |
|  |  | 17 | CSTCS:5150 | 0.572 | 0.118 |
|  |  | 15 | CSTCS:16504 | 0.725 | 0.133 |
|  |  | 15 | CSTCS:9905 | 0.474 | 0.067 |
|  |  | 14 | CSTCS:20125 | 0.496 | 0 |
|  |  | 14 | CSTCS:9906 | 0.593 | 0.071 |
|  |  | 14 | CSTCS:20130 | 0.499 | 0 |
|  |  | 13 | CSTCS:9899 | 0.629 | 0.154 |
|  |  | 13 | CSTCS:18852 | 0.449 | 0 |
|  |  | 12 | PMID:23161681 | 0.781 | 0.333 |
|  |  | 11 | CSTCS:8353 | 0.469 | 0 |
|  |  | 11 | CSTCS:9909 | 0.669 | 0.182 |
|  |  | 11 | CSTCS:3746 | 0.534 | 0.182 |
|  |  | 10 | CSTCS:8360 | 0.528 | 0 |
|  |  | 10 | CSTCS:20127 | 0.549 | 0 |
|  |  | 10 | PMID:19552482 | 0.643 | 0 |
|  |  | 9 | CSTCS:8354 | 0.494 | 0.111 |
|  | **Kme2** | 52 | PMID:30395435 | 0.482 | 0.02 |
|  |  | 20 | CSTCS:5995 | 0.538 | 0.158 |
|  |  | 19 | CSTCS:5153 | 0.595 | 0.158 |
|  |  | 16 | CSTCS:8356 | 0.514 | 0.188 |
|  |  | 15 | CSTCS:5156 | 0.569 | 0.133 |
|  |  | 15 | CSTCS:5154 | 0.502 | 0.067 |
|  |  | 14 | CSTCS:3777 | 0.438 | 0.071 |
|  |  | 13 | CSTCS:3750 | 0.497 | 0 |
|  |  | 8 | CSTCS:8357 | 0.588 | 0 |
|  |  | 7 | PMID:16446289 | 0.478 | 0.143 |
|  |  | 6 | PMID:23161681 | 0.722 | 0.333 |
|  |  | 5 | PMID:26566685 | 0.857 | 0.8 |
|  | **Kme3** | 63 | PMID:30395435 | 0.541 | 0.079 |
|  |  | 24 | CSTCS:7364 | 0.477 | 0.042 |
|  |  | 9 | CSTCS:8358 | 0.383 | 0.111 |
|  |  | 8 | CSTCS:7363 | 0.586 | 0 |
|  |  | 5 | PMID:23161681 | 0.74 | 0.2 |
|  |  | 5 | CSTCS:8359 | 0.364 | 0 |
|  | **Kme** | 1407 | PMID:26750096 | 0.542 | 0.091 |
|  |  | 211 | PMID:25505155 | 0.584 | 0.232 |
|  |  | 166 | PMID:30395435 | 0.484 | 0.066 |
|  |  | 75 | PMID:27577262 | 0.517 | 0.093 |
|  |  | 32 | CSTCS:9897 | 0.606 | 0.188 |
|  |  | 30 | CSTCS:20128 | 0.633 | 0.167 |
|  |  | 26 | PMID:23161681 | 0.606 | 0.308 |
|  |  | 25 | CSTCS:20129 | 0.703 | 0.4 |
|  |  | 24 | CSTCS:7364 | 0.533 | 0.042 |
|  |  | 21 | CSTCS:20132 | 0.683 | 0.381 |
|  |  | 20 | CSTCS:20133 | 0.522 | 0.1 |
|  |  | 20 | CSTCS:5995 | 0.575 | 0.15 |
|  |  | 19 | CSTCS:5153 | 0.469 | 0.053 |
|  |  | 19 | PMID:18247584 | 0.378 | 0.053 |
|  |  | 18 | CSTCS:9896 | 0.432 | 0.111 |
|  |  | 18 | CSTCS:18853 | 0.465 | 0 |
|  |  | 18 | CSTCS:5151 | 0.442 | 0 |
|  |  | 17 | CSTCS:5150 | 0.608 | 0.294 |
|  |  | 17 | CSTCS:16504 | 0.501 | 0.176 |
|  |  | 16 | CSTCS:8356 | 0.596 | 0.312 |
|  |  | 15 | CSTCS:5156 | 0.463 | 0.133 |
|  |  | 15 | CSTCS:9905 | 0.406 | 0 |
|  |  | 15 | CSTCS:5154 | 0.496 | 0.133 |
|  |  | 14 | CSTCS:20125 | 0.618 | 0.286 |
|  |  | 14 | CSTCS:9906 | 0.626 | 0.214 |
|  |  | 14 | CSTCS:20130 | 0.584 | 0.286 |
|  |  | 14 | CSTCS:3777 | 0.482 | 0.143 |
|  |  | 13 | CSTCS:3750 | 0.572 | 0.077 |
|  |  | 13 | CSTCS:18852 | 0.499 | 0.154 |
| **MusiteDeep** | **Kme** | 1866 | PMID:26750096 | 0.693 | 0.3 |
|  |  | 1515 | PMID:25514926 | 0.524 | 0.131 |
|  |  | 500 | PMID:23644510 | 0.515 | 0.118 |
|  |  | 330 | PMID:25505155 | 0.702 | 0.355 |
|  |  | 170 | PMID:30395435 | 0.541 | 0.135 |
|  |  | 106 | CSTCS:20129 | 0.662 | 0.292 |
|  |  | 106 | CSTCS:9897 | 0.662 | 0.292 |
|  |  | 105 | CSTCS:20128 | 0.644 | 0.267 |
|  |  | 96 | CSTCS:20132 | 0.641 | 0.271 |
|  |  | 95 | PMID:27577262 | 0.705 | 0.305 |
|  |  | 89 | PMID:24129315 | 0.7 | 0.348 |
|  |  | 88 | CSTCS:18852 | 0.593 | 0.25 |
|  |  | 83 | PMID:23748837 | 0.689 | 0.277 |
|  |  | 67 | CSTCS:20130 | 0.668 | 0.254 |
|  |  | 59 | CSTCS:20125 | 0.707 | 0.305 |
|  |  | 58 | CSTCS:20131 | 0.645 | 0.276 |
|  |  | 58 | CSTCS:7364 | 0.354 | 0.052 |
|  |  | 55 | CSTCS:20127 | 0.668 | 0.236 |
|  |  | 42 | CSTCS:5995 | 0.586 | 0.071 |
|  |  | 40 | CSTCS:16501 | 0.721 | 0.3 |
|  |  | 40 | CSTCS:16504 | 0.722 | 0.3 |
|  |  | 39 | CSTCS:9906 | 0.668 | 0.308 |
|  |  | 38 | CSTCS:9905 | 0.59 | 0.158 |
|  |  | 38 | PMID:18247584 | 0.438 | 0.053 |
|  |  | 37 | CSTCS:5150 | 0.594 | 0.135 |
|  |  | 36 | CSTCS:5153 | 0.529 | 0.222 |
|  |  | 35 | CSTCS:9896 | 0.462 | 0.114 |
|  |  | 34 | CSTCS:9904 | 0.718 | 0.324 |
|  |  | 32 | CSTCS:3750 | 0.423 | 0.062 |
|  |  | 30 | CSTCS:9899 | 0.704 | 0.267 |
|  |  | 29 | CSTCS:8356 | 0.473 | 0.034 |
|  |  | 28 | CSTCS:5156 | 0.64 | 0.25 |
|  |  | 27 | CSTCS:3777 | 0.539 | 0.037 |
|  |  | 27 | CSTCS:8353 | 0.51 | 0.037 |
|  |  | 25 | CSTCS:5151 | 0.464 | 0.12 |
|  |  | 23 | CSTCS:3746 | 0.616 | 0 |
|  |  | 23 | CSTCS:5154 | 0.45 | 0.13 |
|  |  | 22 | CSTCS:8360 | 0.646 | 0.091 |
|  |  | 22 | PMID:23583077 | 0.448 | 0.091 |
|  |  | 21 | CSTCS:9903 | 0.634 | 0.238 |
|  |  | 19 | CSTCS:8357 | 0.516 | 0.105 |
|  |  | 19 | CSTCS:9909 | 0.674 | 0.211 |
|  |  | 18 | CSTCS:14368 | 0.765 | 0.444 |
|  |  | 18 | CSTCS:3776 | 0.765 | 0.444 |
|  |  | 16 | CSTCS:8354 | 0.713 | 0.25 |
|  |  | 16 | CSTCS:9902 | 0.559 | 0.188 |
|  |  | 15 | CSTCS:5158 | 0.459 | 0.133 |
|  |  | 14 | PMID:23161681 | 0.691 | 0.429 |
|  |  | 13 | PMID:uniprot | 0.667 | 0.385 |

* Sensitivity value when specificity was set 0.9.
