# Supplementary material for: Development of an experiment-split method for benchmarking the generalization of a PTM site predictor: Lysine methylome as an example
Source: PLoS Comput Biol. 2021 Dec 8;17(12):e1009682. doi: 10.1371/journal.pcbi.1009682 (PMC8687584; doi:10.1371/journal.pcbi.1009682)
Supplement: S4 Table — (DOCX) [file pcbi.1009682.s004.docx]

**S4 Table. Prediction performance for CNN_OH_ in terms of experiment-split test.**

| **Modification Type** | **Number** | **Evidence** | **AUC** | **Sn(Sp=0.9)** | **Sn(Sp=0.95)** | **Sn(Sp=0.99)** |
| --- | --- | --- | --- | --- | --- | --- |
| **Kme1** | 216 | PMID:25505155 | 0.809 | 0.431 | 0.25 | 0.093 |
|  | 156 | PMID:23644510 | 0.606 | 0.179 | 0.083 | 0.026 |
|  | 67 | PMID:27577262 | 0.707 | 0.328 | 0.194 | 0.104 |
|  | 52 | PMID:30395435 | 0.577 | 0.154 | 0.019 | 0 |
|  | 50 | PMID:24129315 | 0.843 | 0.66 | 0.46 | 0.18 |
|  | 28 | CSTCS:9897 | 0.929 | 0.75 | 0.607 | 0.321 |
|  | 28 | CSTCS:20129 | 0.956 | 0.893 | 0.75 | 0.607 |
|  | 27 | CSTCS:20132 | 0.882 | 0.815 | 0.556 | 0.333 |
|  | 26 | CSTCS:18852 | 0.906 | 0.692 | 0.615 | 0.385 |
|  | 25 | CSTCS:9896 | 0.833 | 0.64 | 0.56 | 0.48 |
|  | 24 | CSTCS:20128 | 0.836 | 0.583 | 0.417 | 0.167 |
|  | 21 | CSTCS:9906 | 0.663 | 0.333 | 0.286 | 0.143 |
|  | 19 | PMID:23748837 | 0.777 | 0.421 | 0.368 | 0.211 |
|  | 19 | PMID:23583077 | 0.423 | 0.053 | 0.053 | 0 |
|  | 19 | CSTCS:5150 | 0.803 | 0.526 | 0.211 | 0 |
|  | 18 | CSTCS:18853 | 0.89 | 0.778 | 0.611 | 0.444 |
|  | 16 | CSTCS:20133 | 0.901 | 0.75 | 0.75 | 0.438 |
|  | 15 | PMID:18247584 | 0.509 | 0 | 0 | 0 |
|  | 15 | CSTCS:16504 | 0.774 | 0.4 | 0.333 | 0.067 |
|  | 15 | CSTCS:20126 | 0.838 | 0.667 | 0.533 | 0.333 |
|  | 14 | CSTCS:9904 | 0.767 | 0.5 | 0.286 | 0.143 |
|  | 13 | CSTCS:5151 | 0.57 | 0.154 | 0.077 | 0 |
|  | 12 | CSTCS:9905 | 0.597 | 0.167 | 0.083 | 0 |
|  | 12 | CSTCS:20130 | 0.788 | 0.417 | 0.25 | 0.25 |
|  | 11 | CSTCS:16501 | 0.843 | 0.636 | 0.455 | 0.182 |
|  | 11 | CSTCS:20125 | 0.969 | 0.909 | 0.818 | 0.545 |
|  | 10 | CSTCS:3746 | 0.683 | 0.3 | 0.1 | 0 |
| **Kme2** | 56 | PMID:23644510 | 0.669 | 0.25 | 0.161 | 0.071 |
|  | 52 | PMID:30395435 | 0.639 | 0.25 | 0.135 | 0.058 |
|  | 24 | CSTCS:5153 | 0.688 | 0.25 | 0.042 | 0 |
|  | 19 | CSTCS:5154 | 0.596 | 0.105 | 0.105 | 0.053 |
|  | 18 | CSTCS:5995 | 0.457 | 0.056 | 0.056 | 0 |
|  | 16 | CSTCS:3750 | 0.651 | 0.188 | 0.062 | 0.062 |
|  | 14 | CSTCS:8357 | 0.647 | 0.286 | 0.143 | 0.143 |
|  | 14 | CSTCS:3777 | 0.645 | 0.071 | 0.071 | 0.071 |
|  | 12 | CSTCS:5156 | 0.666 | 0.25 | 0.083 | 0 |
|  | 11 | CSTCS:8356 | 0.812 | 0.545 | 0.364 | 0.091 |
|  | 11 | PMID:23161681 | 0.807 | 0.545 | 0.455 | 0.273 |
|  | 10 | PMID:24129315 | 0.64 | 0.5 | 0.5 | 0.5 |
| **Kme3** | 63 | PMID:30395435 | 0.588 | 0.175 | 0.143 | 0.016 |
|  | 48 | PMID:23644510 | 0.699 | 0.229 | 0.146 | 0.021 |
|  | 27 | CSTCS:7364 | 0.8 | 0.519 | 0.444 | 0.407 |
|  | 11 | CSTCS:7363 | 0.767 | 0.455 | 0.455 | 0.091 |
|  | 9 | CSTCS:8358 | 0.83 | 0.444 | 0.444 | 0.111 |
|  | 8 | PMID:24129315 | 0.668 | 0.625 | 0.375 | 0.25 |
|  | 6 | PMID:26750096 | 0.879 | 0.5 | 0.5 | 0.5 |
|  | 5 | CSTCS:8359 | 0.738 | 0.4 | 0.2 | 0 |
|  | 5 | CSTCS:16504 | 0.589 | 0.4 | 0.2 | 0.2 |
| **Kme** | 281 | PMID:23644510 | 0.623 | 0.149 | 0.1 | 0.007 |
|  | 216 | PMID:25505155 | 0.789 | 0.412 | 0.278 | 0.111 |
|  | 166 | PMID:30395435 | 0.583 | 0.133 | 0.066 | 0.006 |
|  | 67 | PMID:27577262 | 0.714 | 0.373 | 0.254 | 0.09 |
|  | 60 | PMID:24129315 | 0.807 | 0.533 | 0.467 | 0.25 |
|  | 57 | PMID:23748837 | 0.753 | 0.368 | 0.263 | 0.14 |
|  | 28 | CSTCS:9897 | 0.928 | 0.679 | 0.536 | 0.429 |
|  | 28 | CSTCS:20129 | 0.974 | 0.929 | 0.857 | 0.571 |
|  | 27 | CSTCS:20132 | 0.869 | 0.741 | 0.556 | 0.333 |
|  | 27 | CSTCS:7364 | 0.817 | 0.407 | 0.259 | 0.074 |
|  | 26 | CSTCS:18852 | 0.867 | 0.654 | 0.615 | 0.423 |
|  | 25 | CSTCS:9896 | 0.829 | 0.64 | 0.6 | 0.52 |
|  | 24 | CSTCS:5153 | 0.839 | 0.417 | 0.167 | 0 |
|  | 24 | CSTCS:20128 | 0.859 | 0.667 | 0.417 | 0.292 |
|  | 21 | CSTCS:9906 | 0.669 | 0.429 | 0.333 | 0.19 |
|  | 20 | PMID:23583077 | 0.355 | 0.05 | 0.05 | 0 |
|  | 19 | CSTCS:5150 | 0.808 | 0.526 | 0.316 | 0.053 |
|  | 19 | CSTCS:5154 | 0.567 | 0.158 | 0.105 | 0 |
|  | 18 | CSTCS:5995 | 0.518 | 0.167 | 0.111 | 0.056 |
|  | 18 | CSTCS:18853 | 0.899 | 0.833 | 0.778 | 0.389 |
|  | 18 | CSTCS:16504 | 0.823 | 0.556 | 0.444 | 0.167 |
|  | 17 | PMID:23161681 | 0.786 | 0.529 | 0.353 | 0.176 |
|  | 17 | PMID:18247584 | 0.578 | 0.118 | 0.118 | 0 |
|  | 16 | CSTCS:3750 | 0.612 | 0.062 | 0.062 | 0 |
|  | 16 | CSTCS:20133 | 0.906 | 0.688 | 0.688 | 0.312 |
|  | 15 | CSTCS:20126 | 0.863 | 0.6 | 0.533 | 0.4 |
|  | 14 | CSTCS:9904 | 0.747 | 0.357 | 0.286 | 0.143 |
|  | 14 | CSTCS:3777 | 0.712 | 0.214 | 0.071 | 0 |
|  | 14 | PMID:uniprot | 0.869 | 0.714 | 0.357 | 0.071 |
|  | 14 | CSTCS:8357 | 0.587 | 0.214 | 0.143 | 0.071 |
|  | 13 | CSTCS:5151 | 0.561 | 0.154 | 0.154 | 0 |
|  | 12 | CSTCS:9905 | 0.605 | 0.25 | 0 | 0 |
|  | 12 | CSTCS:20130 | 0.748 | 0.333 | 0.333 | 0.25 |
|  | 12 | CSTCS:5156 | 0.598 | 0 | 0 | 0 |
|  | 11 | CSTCS:8356 | 0.651 | 0 | 0 | 0 |
|  | 11 | CSTCS:20125 | 0.953 | 0.909 | 0.727 | 0.455 |
|  | 11 | CSTCS:16501 | 0.874 | 0.636 | 0.545 | 0.273 |
|  | 11 | CSTCS:7363 | 0.669 | 0.091 | 0.091 | 0 |
|  | 10 | PMID:16627869 | 0.915 | 0.8 | 0.8 | 0 |
|  | 10 | CSTCS:3746 | 0.765 | 0.3 | 0.1 | 0 |
