# Supplementary figures and images for: Development of an experiment-split method for benchmarking the generalization of a PTM site predictor: Lysine methylome as an example
Source: PLoS Comput Biol. 2021 Dec 8;17(12):e1009682. doi: 10.1371/journal.pcbi.1009682 (PMC8687584; doi:10.1371/journal.pcbi.1009682)

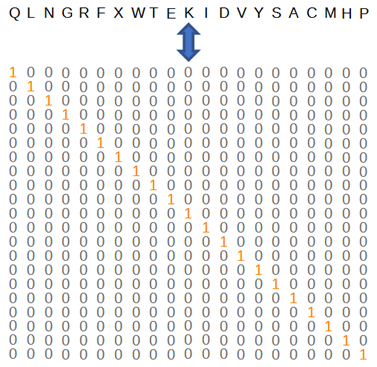

Supplement: S1 Fig — (TIF) [file pcbi.1009682.s005.tif]
